# Intramolecular water-splitting reaction in single collisions of water ions with surfaces†
†Electronic supplementary information (ESI) available: Additional data. See DOI: 10.1039/c6sc05065d



**DOI:** 10.1039/c6sc05065d

**Published:** 2017-01-18

**Authors:** Yunxi Yao, Konstantinos P. Giapis

**Affiliations:** a Division of Chemistry and Chemical Engineering , California Institute of Technology , Pasadena , California 91125 , USA . Email: giapis@cheme.caltech.edu

## Abstract

We report the direct formation of H_2_ and O pair ions through single collisions of water ions with metal surfaces at hyperthermal energies. This unusual intramolecular reaction proceeds also for heavy and semi-heavy water, producing molecular D_2_ and HD ions. The selectivity of this water splitting channel is estimated at being between 9 and 13% *versus* complete dissociation. The collision kinematics support the hypothesis of a water molecule colliding with a single surface atom, thereby forming an excited precursor (Rydberg?) state, which dissociates subsequently to form the molecular hydrogen ion with high kinetic energy. Inelastic energy loss considerations yield an estimate for the energy of the excited precursor state of ∼7 eV and ∼11 eV at low and high incidence energies. These energies are close to the Ã state (^1^B_1_, 7.5 eV) and B state (^1^A_1_, 9.7 eV) of excited water (Rydberg states).

## Abstract 2

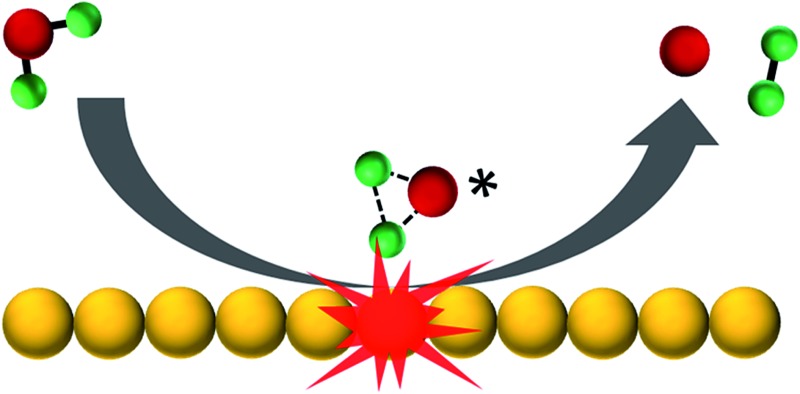
Direct water splitting into molecular hydrogen and atomic oxygen is demonstrated through single collisions of water ions with generic surfaces at hyperthermal energies.

## Introduction

Water dissociation is of paramount importance in the cosmos, the atmosphere, the earth, and life. It may occur in multiple ways, with thermal, photonic, electronic, or kinetic energy provided to the water molecule. One way that leads to the production of molecular hydrogen, known as water splitting, is actively pursued by indirect photo- and electro-catalytic schemes.

Three elementary dissociation processes are possible for neutral water, with the indicated dissociation energies:^1,2^

I H_2_O → OH + H (5.1 eV)

II H_2_O → O + H_2_ (5.0 eV)

III H_2_O → O + 2H (9.5 eV)

Channel (I) occurs predominantly and has been widely studied in heterogeneous catalysis under thermal conditions, owing to its importance in steam reforming and water gas-shift reactions.^3–6^ Channel (III) represents the complete dissociation of water into its constituent atoms, typically occurring at very high temperatures or in collisions with high-energy photons or electrons. Although channel (II) is energetically comparable to channel (I), direct formation of molecular hydrogen is extremely rare. For example, production of H_2_^+^ by electron impact dissociation of gaseous water has a reaction cross-section of 8 × 10^–20^ cm^2^ at an electron energy of 100 eV, too small to be significant.^1^ However, channel (II) can become important following internal excitation of the water molecule to form higher-lying Rydberg states, which may dissociate into H_2_ (X^1^Σ+g) and O (^3^P_g_ or ^1^D_g_). This process was indeed found to occur in water photolysis with vacuum-ultraviolet (VUV) photons with 10.2 eV energy (second absorption band), which has been attributed to the formation of the singlet B (^1^A_1_) state of water.^7,8^ This unusual intramolecular reaction, which produces a diatomic molecule from the end atoms of a triatomic parent molecule, has also been observed in other systems.^9^ For example, molecular O_2_ has been produced directly from CO_2_ in the gas-phase by VUV photolysis and also by dissociative electron attachment with an electron energy between 15.9 and 19 eV.^10,11^ Such reaction pathways are not accessible using thermal energy; rather, they require photonic, electronic, or even nuclear excitation means. We show here another unexplored way to drive such reactions by means of gas-surface collisions at hyperthermal incidence energies (60–300 eV). In particular, we demonstrate the direct production of molecular hydrogen in single-collisions of energetic water ions with Au surfaces. Intramolecular excitation induced by surface collisions may offer a simpler and more efficient way to energize molecules and drive such reactions, especially in astrophysical environments, where energetic ions may be found. Indeed, water ions have been detected in the inner coma of comet 67P, formed by photoionization, which are then picked up in the extended coma and accelerated by solar wind towards the nucleus surface with kinetic energies between 120 and 800 eV.^12,13^ When water ions collide with the nucleus surface and dust grains, containing silicates and iron oxides, molecular hydrogen is produced *via* the reported intramolecular water-splitting reaction (see the ESI, Fig. S5†). As demonstrated below, the dynamic production of H_2_ from water ion collisions with surfaces occurs with high yield and, thus, it may contribute significantly to the formation of molecular hydrogen under cometary and interstellar conditions.^14^

## Experimental

The scattering experiments were carried out in a home-built, ultra-high vacuum (UHV) scattering system, coupled to an ion beam-line, described elsewhere.^15,16^ The ions were produced in an inductively-coupled plasma reactor, with tunable plasma potential. Ions were extracted, collimated, and launched into the ion beam line, held typically at –15 kV, then underwent magnetic mass filtering. Isotopically-pure ion beams of H_2_O, HOD, and D_2_O, with currents between 2 and 5 μA, were directed towards a pre-cleaned, polycrystalline Au surface, held at room temperature. The angle of incidence was set at 45° and the scattered products were detected at a 45° angle of exit. The ion beam energy was tuned by adjusting the plasma bias. All scattered products were mass-resolved using a triply-differentially pumped mass spectrometer (Extrel QPS), and their kinetic energy was measured using a home-built and calibrated 90° sector energy analyzer with a pass energy of 15 eV. A channel electron multiplier was used to detect positive or negative ions, biased accordingly. Fast neutrals cannot be detected in this system. All signals were normalized with a beam current at a corresponding energy.

## Results and discussion

**Fig. 1:**
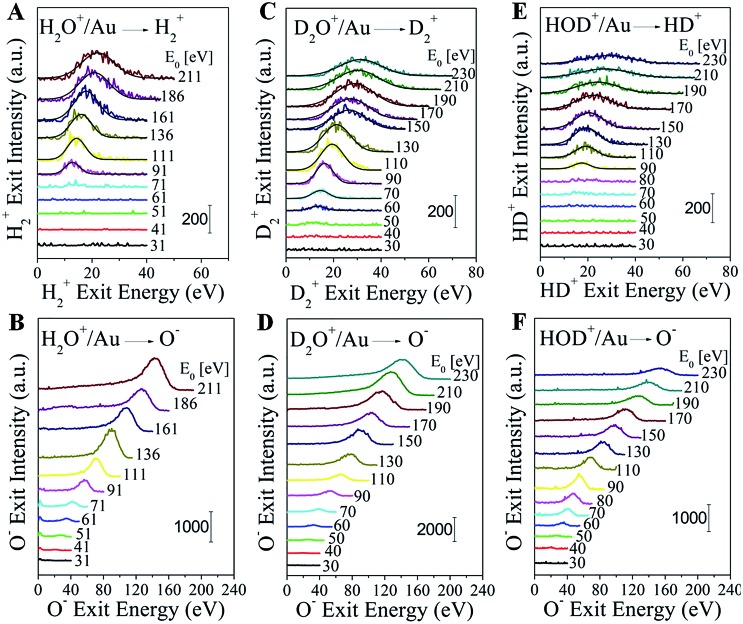
Direct formation of molecular H_2_^+^, D_2_^+^ and HD^+^ in water–ion collisions on gold. Energy distributions of (A) H_2_^+^ and (B) O^–^ from H_2_O^+^/Au; (C) D_2_^+^ and (D) O^–^ from D_2_O^+^/Au; (E) HD^+^ and (F) O^–^ from HOD^+^/Au as a function of the corresponding product energy. Distributions are shown for multiple incidence energies (*E*_0_) of the corresponding water ion.

Water–ion bombardment of a clean Au surface, denoted as H_2_O^+^/Au, results in the following scattered products: H_2_^+^, H^+^, OH^+^, OH^–^, O^+^, and O^–^, appearing at different energy thresholds (Fig. S1†). H_2_O^+^ ions are also detected, but only at a low incidence energy (≤135 eV). Surprisingly, fast H_2_^+^ molecular ions are observed exiting the surface at a beam energy *E*_0_ ≥ 90 eV (Fig. 1A). The H_2_^+^ peak position shifts monotonically with the H_2_O^+^ incidence energy and the peak also broadens. This energy dependence rules out surface sputtering as the origin of the fast H_2_^+^ product, since sputtering peaks do not shift much with beam energy. Furthermore, sputtering requires that H atoms or H_2_ molecules be present on the Au surface, a condition hindered by their tendency to desorb promptly from Au even at temperatures below 200 K.^17^ For the same reason, pre-dissociation channels, followed up by H abstraction reactions, are also excluded. Gas-phase reactions with background hydrogen should be extremely rare under the ultrahigh vacuum (UHV) conditions of the experiment (1–2 × 10^–8^ Torr). The only remaining way to explain the mysterious H_2_^+^ peak is directly *via* channel (II) from surviving or neutralized water ions after collision with the Au surface. We conjecture that this process occurs through a highly-excited Rydberg state formed during the hard collision. If the process is similar to water photolysis at high photon energies, a corresponding atomic oxygen fragment should be detectable. We show below that this fragment is O^–^, co-produced in a pair-ion formation scheme and captured in the energy spectra of Fig. 1B.

In order to further confirm the direct formation of molecular hydrogen and exclude any possibility of background contamination, heavy water (deuterium oxide) was used to perform D_2_O^+^ scattering on Au. The scattering behavior is similar to that seen with normal water. D_2_O^+^/Au produces the following ion fragments: D_2_^+^, D^+^, OD^+^, OD^–^, O^+^, and O^–^ (Fig. S2†). No HD^+^, nor HD^–^, nor any H^+^ are detected, despite the ubiquitous presence of hydrogen in the UHV background. D_2_^+^ becomes detectable at an even lower threshold of ∼60 eV than that for detecting H_2_^+^ in H_2_O^+^/Au (Fig. 1C). Note that O^–^ appears simultaneously with D_2_^+^ (Fig. 1D). The exit energies of both ion fragments increase monotonically with the D_2_O^+^ incidence energy.

The final proof for collisional water splitting is obtained by performing HOD^+^ scattering on Au (Fig. S3†). In addition to all other products, of note here are the detected H^+^, D^+^ and HD^+^ ion exits. The absence of D_2_^+^ excludes the possibility that fast molecular hydrogen can be formed by surface recombination or abstraction reactions with beam delivered D sticking at the surface (Fig. S4†). The HD^+^ and O^–^ products that are detected (Fig. 1E and F) exhibit striking similarity to the behavior of H_2_^+^, D_2_^+^ and O^–^ in the H_2_O^+^/Au and D_2_O^+^/Au experiments.

**Fig. 2:**
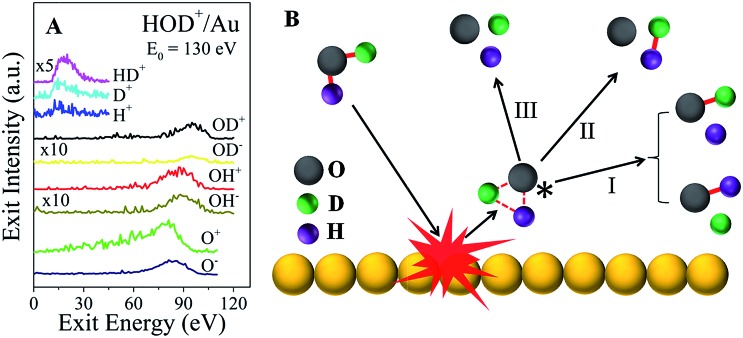
Excitation and dissociation of molecular water in single-collisions with gold at hyperthermal energies. (A) Energy distributions of the dissociation products, HD^+^, H^+^, D^+^, OD^+^, OD^–^, OH^+^, OH^–^, O^+^, and O^–^, observed from HOD^+^/Au at an incidence energy of 130 eV. (B) Schematic depiction of the proposed excited molecular-water state, formed in a single-collision of energetic HOD^+^ with a Au surface atom. The transient precursor state dissociates subsequently to form a multitude of daughter fragments, including molecular HD.

In addition to the fast molecular hydrogen ions, there is a multitude of dissociation fragments for each of the three water ions. Typical survey spectra of the ion products that are detected from HOD^+^/Au are presented in Fig. 2A for *E*_0_ = 130 eV. At this incidence energy, no molecular water ions survive. Note that some fragments appear only as positive ions, while others appear with both polarities. All of these and any additional neutral fragments may be explained simply by a common transient precursor, which we propose to be a higher lying Rydberg state of water, excited during a single-collision of the water ion with the Au surface. For brevity, we will only discuss here the HOD^+^/Au results. The formation of the transient HOD* Rydberg state and its subsequent three dissociation channels are schematically depicted in Fig. 2B. Some of these fragments may be produced as ion pairs during the dissociation, *e.g.* (HD^+^, O^–^), (H^+^, OD^–^), *etc.*, or they may be ionized afterwards *via* charge exchange with the surface. Depending on the incidence energy, a number of higher lying Rydberg states may be accessible, which will influence the dissociation branching ratios. We note here that the production of molecular hydrogen is generic to the surface used (Fig. S5†). Apparently, the role of the surface is to cause molecular excitation with no subsequent participation. The common precursor hypothesis explains the energy difference between the OH and OD fragments, seen in Fig. 2A, which must be produced by breaking of the HO–D and H–OD bonds,^18^ respectively. Momentum conservation for the delayed fragmentation of HOD* suggests that OD should possess more kinetic energy than OH, as is indeed observed. The difference in atomic mass between H and D breaks the molecular symmetry, which raises the possibility that bond dissociation within the HOD* molecule may be selective. Indeed, at *E*_0_ = 130 eV, the OH signal intensity is larger than that of OD for both polarities.

**Fig. 3:**
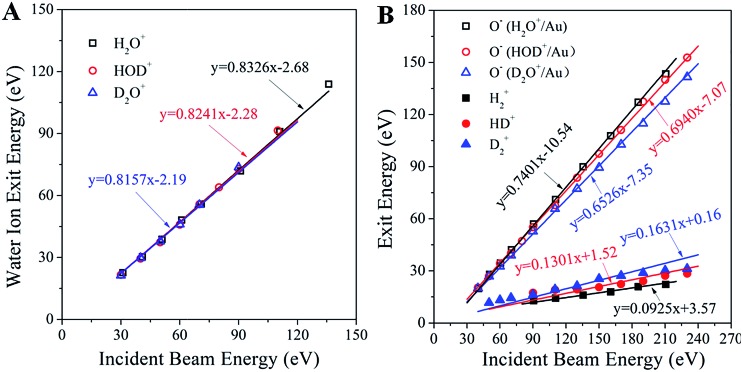
Kinematics of molecular hydrogen formation in water–ion collisions with gold. (A) Exit energies of the molecular water ions H_2_O^+^, HOD^+^ and D_2_O^+^ as a function of the corresponding water ion incidence energy. The three solid lines (nearly overlapping) are linear fittings with fixed slopes, equal to the kinematic factor predicted from the BCT for a single collision between the corresponding water ion and an Au surface atom at a deflection angle of 90°. The correlation factor (*R* ^2^) for all fittings is better than 0.995. (B) Exit energies of the H_2_^+^, HD^+^, D_2_^+^, and the partner O^–^ product ions from scattering and dissociation of H_2_O^+^, HOD^+^ and D_2_O^+^ on Au, as a function of the corresponding water ion incidence energy. The points are experimentally obtained peak energies, inferred from Fig. 1. The solid lines are linear fittings with fixed kinematic factors, calculated from energy conservation by assuming that the ion products are fragments of the excited states of the corresponding water molecules, which dissociate after surface collision. Note that the sum of the slopes of each product ion pair equals the slope of the corresponding parent ion.

Analysis of the kinematics of each ion product is key to understanding the collisional interactions. Fig. 3A illustrates the dependence of the surviving water–ion exit energy as a function of the incidence energy for all of the three water ions studied. The data are fitted linearly with fixed slopes of 0.8326, 0.8241 and 0.8157 for H_2_O^+^, HOD^+^ and D_2_O^+^, respectively, which are the exact kinematic factors predicted by the binary collision theory (BCT)^19,20^ for water scattering on Au as a whole molecule. The remarkably good fit (correlation coefficient better than 0.995 for all fits) suggests strongly that the detected signals are due to single collisions with surface atoms. The intercept, in the range of –2.19 to –2.68 eV, represents the inelastic energy loss occurring during the surface scattering process. This energy loss is too small to account for the production of the hypothesized Rydberg state or the re-ionization of a neutralized water molecule, confirming that the detected water ion survives the collision. Fig. 3B shows the measured exit energy of the H_2_^+^, D_2_^+^and HD^+^ product ions, and the corresponding pair ion O^–^, as a function of the incident beam energy. First, we note that at any given incidence energy, D_2_^+^ has the highest exit energy, followed by HD^+^ and H_2_^+^, while the corresponding O^–^ ions show the opposite trend with the lowest exit energy for O^–^ from D_2_O^+^/Au. Second, it is clear from these plots that all of the data sets can be fitted linearly. Can the slopes (*i.e.* kinematic factors) of these lines be predicted?

Upon approaching the Au surface, most of the incoming water ions are expected to become neutralized before the hard collision.^21^ Surface-collisions at hyperthermal energies may induce electron promotions leading to excited states.^22^ The energy required for promotion must originate in the incidence energy and should result in a projectile exiting the surface with less kinetic energy than that predicted by the BCT. The difference between these two energies, termed inelasticity, is a fixed amount of energy relating to the excited state produced.^22^ As noted earlier, the inelasticity of the molecular water ion exit (∼2 eV) was too small to account for the formation of a Rydberg state. The hypothesized excited water precursor state (*e.g.* HOD*) should have a larger inelasticity, at least 7 to 10 eV, based on photo-excitation requirements.^23^ Thus, the excited precursor state must be formed differently, possibly through resonant neutralization and electronic promotion due to exclusion.^21,22^ Given its transient nature, a possible delayed fragmentation of the precursor state would provide a way to estimate its inelasticity by looking at the dissociation products (*e.g.* HD^+^, O^–^). In that case, the kinematics of producing the excited HOD* should be identical to that of producing the surviving HOD^+^, albeit with a substantially different inelasticity. This argument makes it possible to determine *a priori* the kinematic factors of the dissociation products H_2_^+^, HD^+^, D_2_^+^, and the corresponding O^–^ pair ion, from the mass-weighted BCT kinematic factor of the observed corresponding water ion. We thus obtain kinematic factors of: 0.0925, 0.1301 and 0.1631 for H_2_^+^, HD^+^ and D_2_^+^, respectively, and 0.7401, 0.6940 and 0.6526 for the corresponding O^–^ ion exits. As can be seen in Fig. 3B, these calculated slopes provide remarkably good linear fittings of the kinematic data, even at higher incidence energies where water ions are no longer detected. This finding supports the conjecture that the fast molecular hydrogen originates from an excited precursor state undergoing delayed fragmentation. The positive intercepts, obtained from the linear fittings of the H_2_^+^, HD^+^ and D_2_^+^ ion exit energies, suggest that the scattered molecular hydrogen ions gain energy upon dissociation of the excited state. The negative intercepts for the O^–^ ion exits indicate inelastic energy losses, notably larger than those of the corresponding water ion that survives the collision. Since O^–^ is the larger fragment, its inelasticity should be related to the formation energy of the excited precursor state, though the exact relationship has not yet been established. The excited precursor is also responsible for the formation of other dissociation products (*i.e.* OH^±^, OD^±^, H^+^, O^+^), whose kinematics can be predicted by similar considerations with few exceptions (Fig. S6†). The ability to describe the kinematics of so many fragments strongly supports the common precursor hypothesis.

**Fig. 4:**
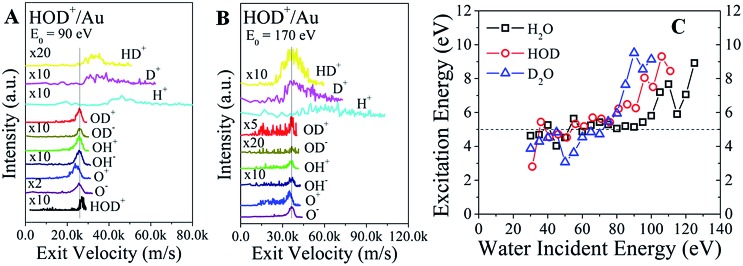
Velocity plots for ion products from water ion scattering on gold. The HOD^+^ incidence energies are (A) 90 eV and (B) 170 eV. Peak alignment in velocity space, shown by the vertical line, indicates which ion fragments originate from a common parent molecule. Note that O^+^ is slower, and the surviving HOD^+^ is faster than the common velocity of OH^±^, OD^±^, and O^–^. (C) Estimated excitation energy of the excited water precursor as a function of incidence energy. The kinetic energy of the excited state is calculated from the sum of the corresponding energies of its dissociated daughter ions. Then, the excitation energy can be obtained from the difference between the energy of the surviving water ion minus that of the excited state.

The final confirmation for the existence of a common precursor comes from a comparison of the product velocities. Since the kinematic energies are much larger than the bond energies, the mean velocities of the heavier fragments should be comparable to the mean velocity of the scattered parent. Indeed, the exit velocities of OD^+^, OD^–^, OH^+^, OH^–^, and O^–^ line up very well, as shown in Fig. 4A for HOD^+^/Au at *E*_0_ = 90 eV. The surviving HOD^+^ is faster than these fragments, as expected from the inelasticity argument above. Remarkably, HD^+^, D^+^, and H^+^ ions are even faster than the water ions, consistent with the dissociation of excited molecules containing hydrogen.^24^ That is, the bond energy of the excited precursor state is channelled more efficiently into lighter fragments. O^+^ is always slower than O^–^, suggesting that it does not originate from the same precursor. At the higher incidence energy of 170 eV (Fig. 4B), HOD^+^ ions are no longer detected. Now, the exit velocity of HD^+^ lines up better with those of OD^±^, OH^±^ and O^–^, as expected from the common precursor hypothesis. However, the HD^+^ peak is very broad, suggesting that its velocity may be influenced by other intramolecular energy partitioning effects. The results for H_2_O^+^/Au and D_2_O^+^/Au (Fig. S7†) indicate similar trends.

The kinetic energy of the excited precursor state can be calculated from the energies of the daughter ions. Then, the difference in energy between the surviving water ion and the excited precursor state may be a proxy for the (electronic?) excitation energy required to produce the excited state. These energy differences for each water ion are plotted in Fig. 4C as a function of the incidence energy. At low energy (*E*_0_ < 80 eV), the calculated excitation energies appear constant at ∼5 eV for all water ions. With increasing energy, the excitation energies jump abruptly to ∼9 eV. Adding the ∼2 eV inelasticity for the surviving water ions (Fig. 3A), the actual excitation energy for the excited precursor state becomes ∼7 eV *vs.* ∼11 eV at low *vs.* high incidence energies. Remarkably, these energies are close to the Ã state (^1^B_1_, 7.5 eV) and B state (^1^A_1_, 9.7 eV) of excited water (Rydberg states).^23^ The Ã state is a repulsive state, which mainly accounts for water dissociation *via* channel (I) at low incidence energies. Higher lying states, such as the B state, should be accessible at incidence energies above a threshold (≥100 eV), where excitation energy appears to be enough for complete dissociation, or direct molecular hydrogen production.

The energy required to form the putative excited Rydberg state cannot possibly originate in surface charge transfer during the collision. Charge transfer at lower collision energies can lead to electronically non-adiabatic processes that promote chemistry.^25^ The intramolecular reaction observed here has a very high kinetic energy threshold (∼100 eV), suggesting that the electronic states of the impinging water ion or molecule must overlap sufficiently with the target atom states to induce electronic promotion in the former. This is consistent with the relative insensitivity of the scattering dynamics observed on the nature of the surface: H_2_^+^ is produced on Au, Pt (not shown), Si-native oxide, oxidized Fe, and oxidized Ni. Although charge transfer at high collision energies may not influence reactivity, it could affect the charge polarity of the scattered products as follows. Given the violence of the collision, the excited state should be relatively short lived. Dissociation of the rebounding molecule near the surface allows the fragments to interact electronically with extended surface states. Charge transfer is possible and will determine whether the products will remain neutral or appear as positive or negative ions. The high work-function metals Au and Pt favor the production of positive ions, yielding only H_2_^+^. In contrast, insulating surfaces, such as SiO_*x*_, readily yield both polarities: H_2_^+^ and H_2_^–^ are both observed (Fig. S5†).

The selectivity of the intramolecular reaction is crucial for impact. In our experiments, channel (II) is activated at intermediate incidence energies, where channels (I) and (III) may also be active. However, when *E*_0_ ≥ 185 eV, only channels (II) and (III) compete, rendering it possible to compare hydrogen production between the channels. By making certain assumptions about ionization and detection efficiencies between molecular and atomic hydrogen ions (Fig. S8†), we have estimated the selectivity of channel (II) to be between 9 and 13% *vs.* complete decomposition. This conservative estimate is consistent with photolysis experiments, reporting values between 6 and 25% depending on photon energy.^8,26^

## Conclusions

Collisional excitation of molecules upon surface impact at hyperthermal incidence energies permits access to exotic molecular configurations that may react in surprising ways. Such interactions are generic to both the chemical identity of the energetic molecule and the nature of the surface, thus offering a simpler way to populate excited states and study their reaction dynamics as compared to extreme electromagnetic, electronic, or nuclear excitation means. One such collisionally-activated intramolecular reaction channel was demonstrated for water molecules, which dissociate promptly into molecular hydrogen and atomic oxygen following a single hyperthermal collision with a metal surface. This unusual water-splitting reaction implies that intramolecular bond rearrangement is possible in a highly-excited Rydberg state. The inelastic energy loss associated with this reaction pathway was estimated to be close to the excitation energy of the first or second Rydberg state of water for low or high incidence energies, respectively.
