# Supplementary material for: Intramolecular water-splitting reaction in single collisions of water ions with surfaces
Source: Chem Sci. 2017 Jan 18;8(4):2852–8. doi: 10.1039/c6sc05065d (PMC5427682; doi:10.1039/c6sc05065d)
Supplement: Supplementary file 1 [file SC-008-C6SC05065D-s001.pdf]

Electronic Supplementary Material (ESI) for Chemical Science.
This journal is © The Royal Society of Chemistry 2017

Supporting Information

Fig. S1-8

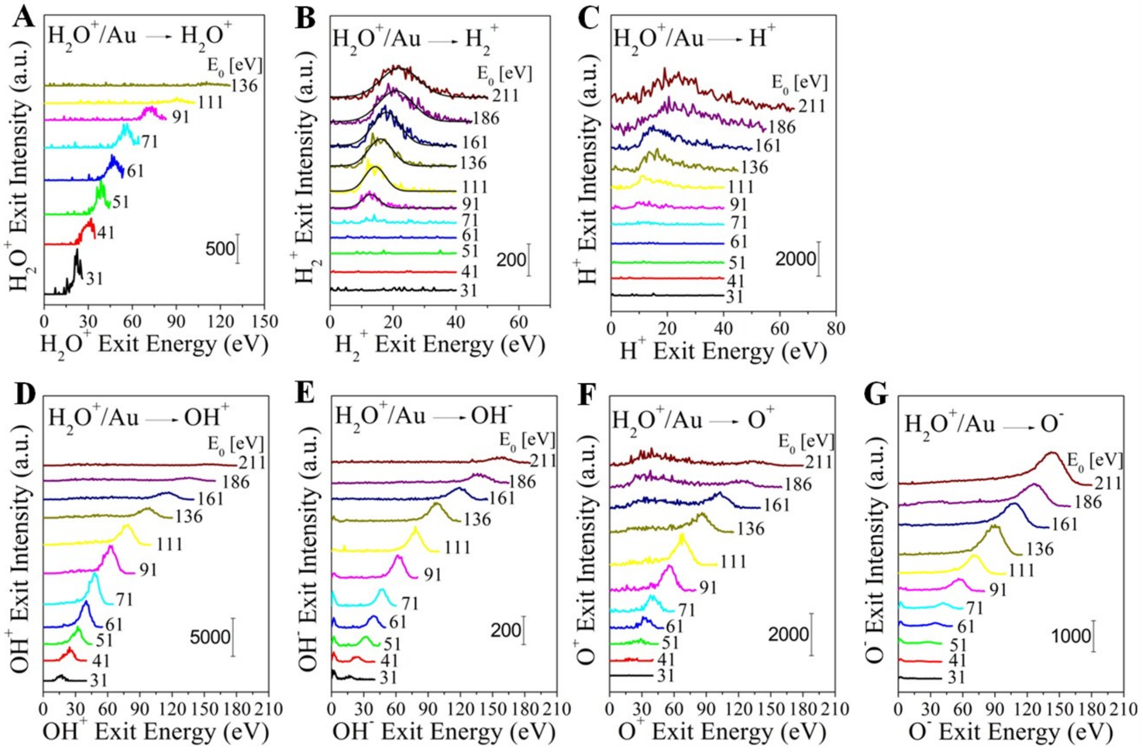

Fig. S1. Energy distributions of all scattered products detected from H_2O^+/Au. Each panel presents data for multiple incident H_2O^+ energies as indicated. The O^+ energy spectra in panel F show a broad low energy peak around 20 eV for incidence energies above 150 eV due to surface sputtering.

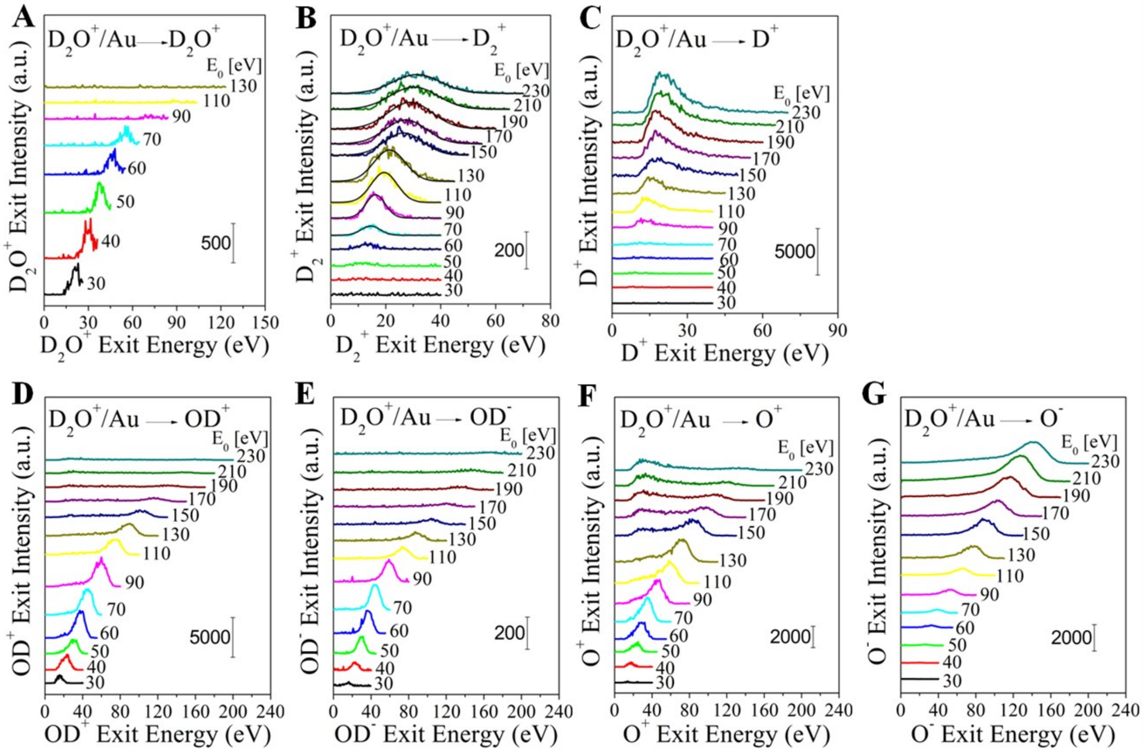

Fig. S2. Energy distributions of all scattered products detected from D_2O^+/Au. Each panel presents data for multiple incident D_2O^+ energies as indicated.

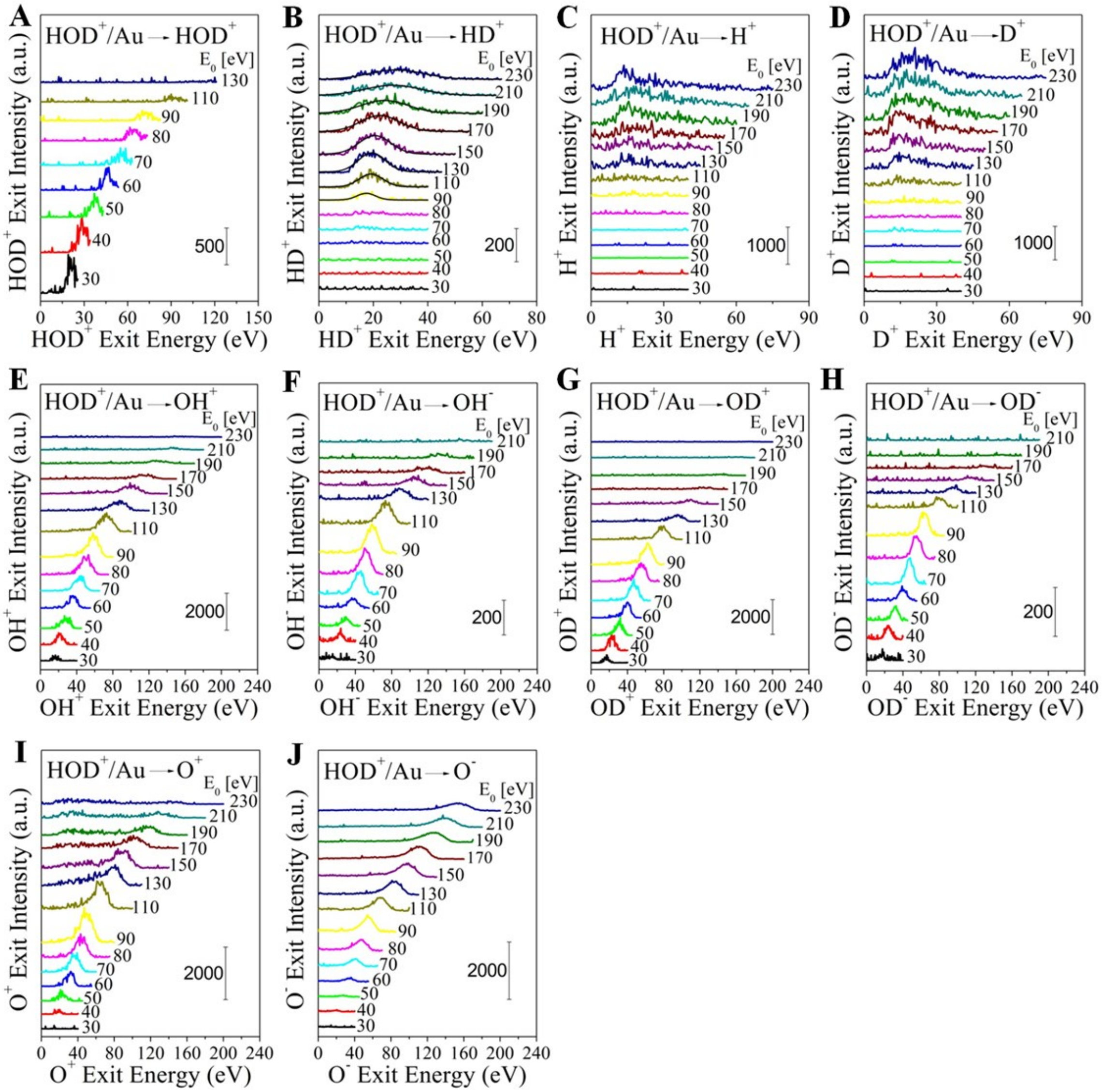

Fig. S3. Energy distributions of all scattered products detected from HOD^+/Au. Each panel presents data for multiple incident HOD^+ energies as indicated.

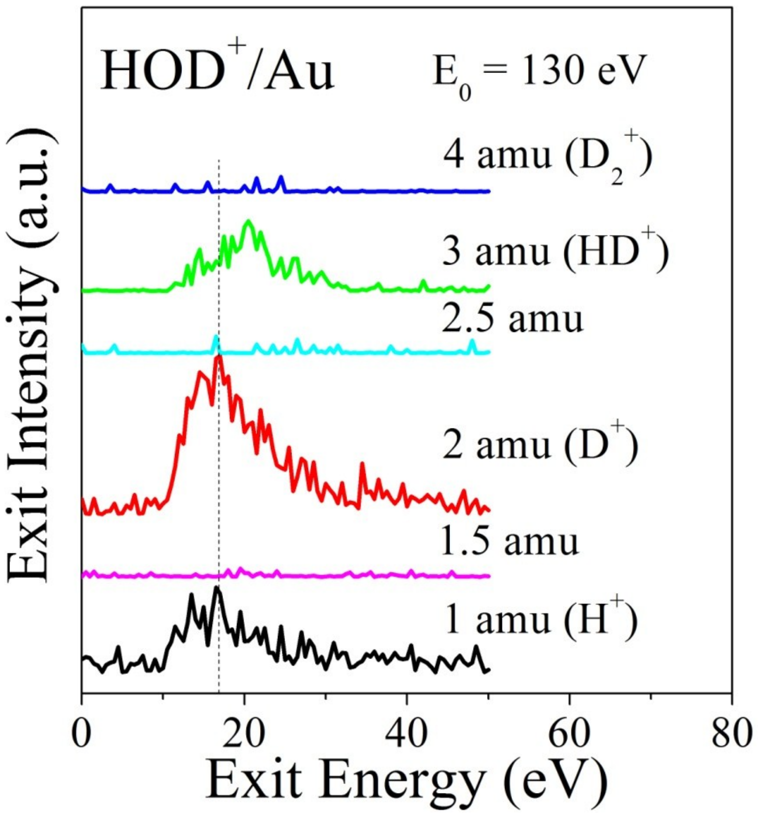

Fig. S4. Hydrogen products from HOD^+/Au at E_0=130eV. Only H^+, D^+ and HD^+ ion exit products are detected. The absence of D_2^+ signal suggests that neither sputtering nor abstraction reactions with adsorbed D can take place. Thus, the fast HD^+ product cannot originate from a surface recombination reaction. The absence of signal at mass spectrometer setting of 1.5 amu and 2.5 amu confirms that the mass resolution is set high enough to prevent interference between adjacent masses.

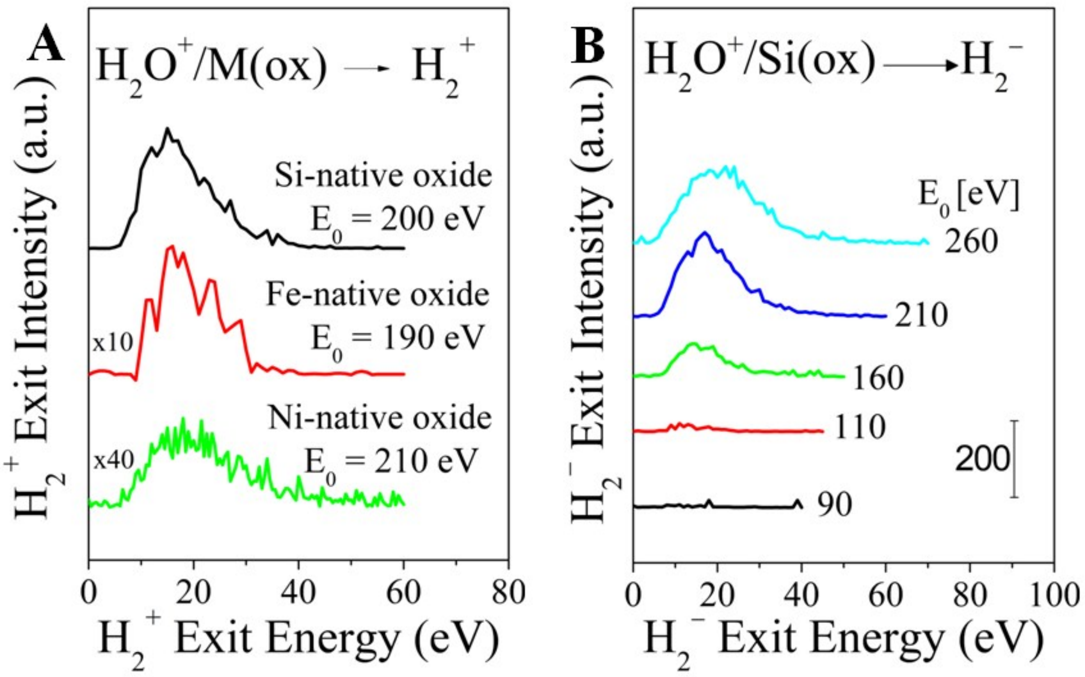

Fig. S5. (A) Molecular H_2^+ production in water ion collisions with oxidized Si, Fe, and Ni. The scattering experiments are identical to those on Au. Energy spectra are shown for representative H_2O^+ incident energies as indicated for each surface. (B) Molecular H_2^- production in water ion collisions with oxidized Si. Unlike scattering on metal surfaces, molecular hydrogen is detected in both polarities in H_2O^+ collisions with oxidized Si.

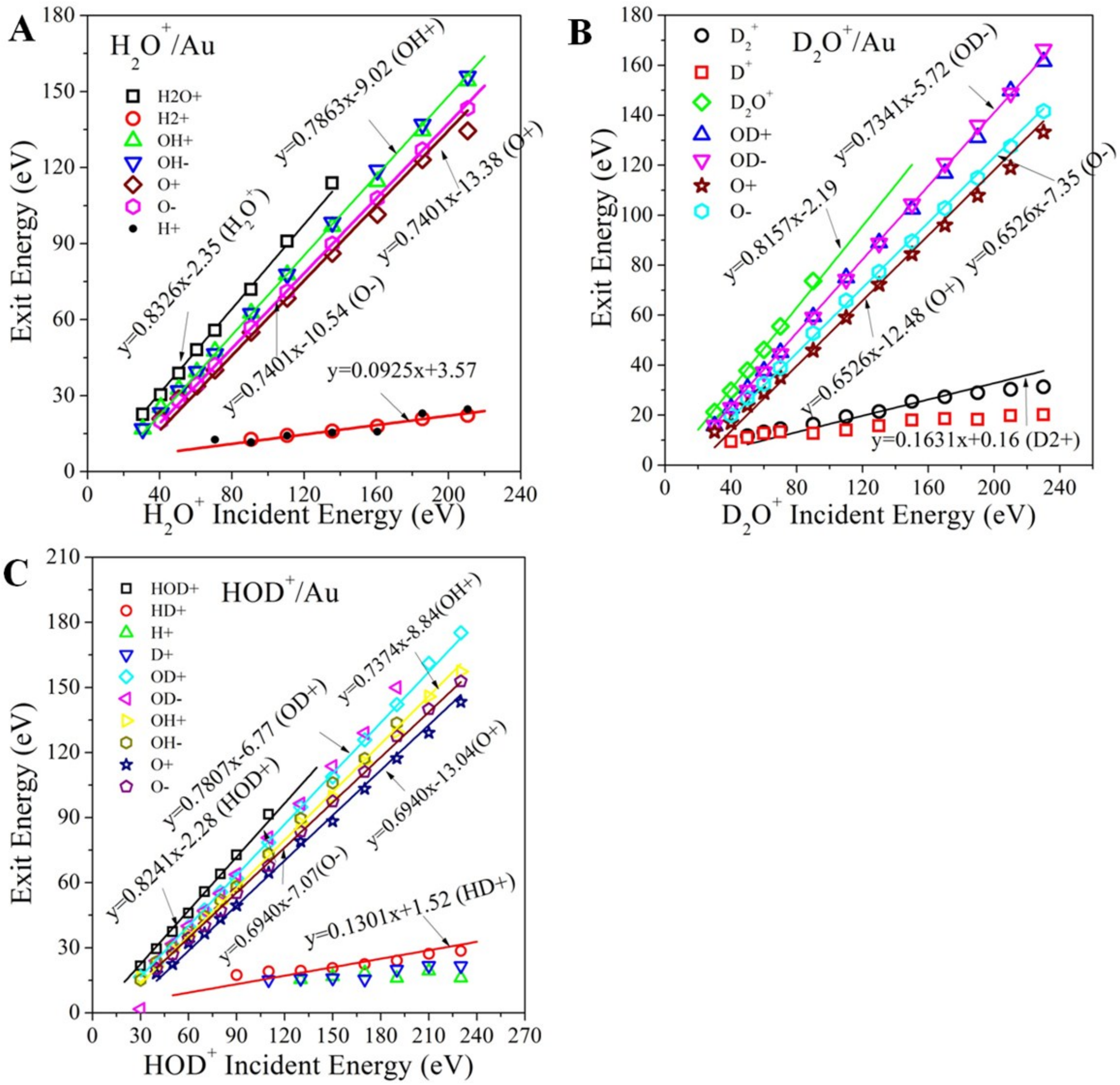

Fig. S6. The complete kinematics of water ion scattering on Au. (A) H_2O^+, (B) D_2O^+, (C) HOD^+. The solid lines are linear fittings with the kinematic factors calculated either from BCT for surviving water ions or by assuming that the scattered ion products originate from the fragmentation of a common precursor molecule, which dissociates after the surface collision.

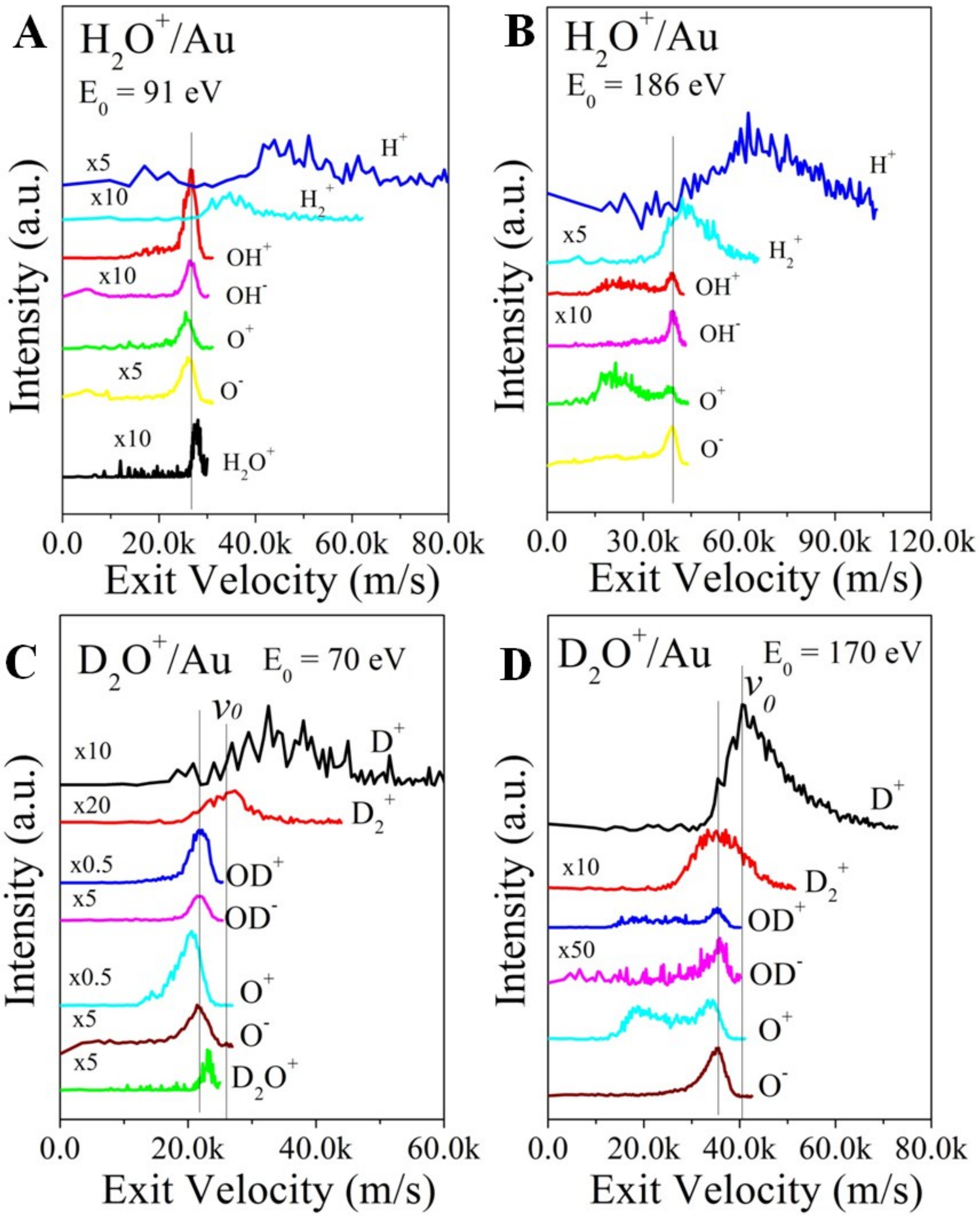

Fig. S7. Velocity plots of scattered ion products from H_2O^+/Au and D_2O^+/Au. The H_2O^+ incident energies are: (A) 91 eV and (B) 186 eV. Peak alignment in velocity space, shown by the vertical line, indicates which ion fragments originate from a common parent molecule. Note that O^+ is slower, and the surviving H_2O^+ is faster than the common velocity of OH^+, OH^-, and O^-. The D_2O^+ incident energies are (C) 70 eV and (D) 170 eV. The D_2O^+/Au scattered fragments behave is similarly to those of H_2O^+/Au.

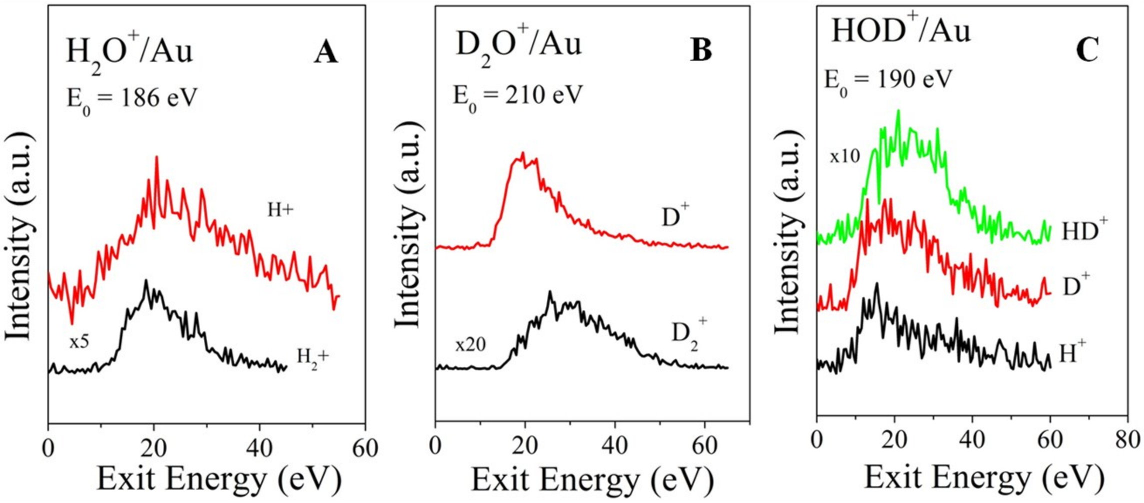

Fig. S8. Estimation of the selectivity for direct molecular hydrogen formation. (A) H^+ and H_2^+ from H_2O^+/Au at E_0=186eV. (B) D^+ and D_2^+ from D_2O^+/Au at E_0=210eV. (C) H^+, D^+ and HD^+ from HOD^+/Au at E_0=190eV. The partial dissociation of water is negligible at the selected incident beam energies, so there are no OH or OD peaks. Therefore, there are only two competing reactions: channel (II) vs. channel (III). Assuming that atomic and molecular hydrogen ions form with the same probability and are detected with the same efficiency, the selectivity of channel (II), $S(X_n)$, can be estimated from integrated signal intensities--$I(X_n)$, X=H,D,HD and n=1,2--as follows: $S(H_2)=I(H_2)/[I(H_2)+I(H)/2)]$; $S(D_2)=I(D_2)/[I(D_2)+I(D)/2]$; $S(HD)=I(HD)/[I(HD)+I(D)/2+I(H)/2]$. The calculated selectivities are: $S(H_2)$= 13.3%; $S(D_2)$=9.2%; S(HD)=12.1%. These values may be conservative estimates, given that atomic hydrogen has lower ionization energy (13.6 eV) than molecular hydrogen (15.4 eV), which may result in a higher formation probability for the former.
